# Supplementary material for: Age-seroprevalence curves for the multi-strain structure of influenza A virus
Source: Nat Commun. 2021 Nov 18;12:6680. doi: 10.1038/s41467-021-26948-8 (PMC8602397; doi:10.1038/s41467-021-26948-8)
Supplement: Supplementary file 3 — Reporting Summary [file 41467_2021_26948_MOESM3_ESM.pdf]

nature research

Corresponding author(s): Maciej F Boni
Last updated by author(s): 10/7/2021

Reporting Summary

Nature Research wishes to improve the reproducibility of the work that we publish. This form provides structure for consistency and transparency in reporting. For further information on Nature Research policies, see our Editorial Policies and the Editorial Policy Checklist.

Statistics

For all statistical analyses, confirm that the following items are present in the figure legend, table legend, main text, or Methods section.

n/a	Confirmed	
☐	☒	The exact sample size (*n*) for each experimental group/condition, given as a discrete number and unit of measurement
☐	☒	A statement on whether measurements were taken from distinct samples or whether the same sample was measured repeatedly
☒	☐	The statistical test(s) used AND whether they are one- or two-sided<br>*Only common tests should be described solely by name; describe more complex techniques in the Methods section.*
☒	☐	A description of all covariates tested
☐	☒	A description of any assumptions or corrections, such as tests of normality and adjustment for multiple comparisons
☐	☒	A full description of the statistical parameters including central tendency (e.g. means) or other basic estimates (e.g. regression coefficient) AND variation (e.g. standard deviation) or associated estimates of uncertainty (e.g. confidence intervals)
☒	☐	For null hypothesis testing, the test statistic (e.g. *F*, *t*, *r*) with confidence intervals, effect sizes, degrees of freedom and *P* value noted<br>*Give P values as exact values whenever suitable.*
☒	☐	For Bayesian analysis, information on the choice of priors and Markov chain Monte Carlo settings
☒	☐	For hierarchical and complex designs, identification of the appropriate level for tests and full reporting of outcomes
☐	☒	Estimates of effect sizes (e.g. Cohen's *d*, Pearson's *r*), indicating how they were calculated

Our web collection on statistics for biologists contains articles on many of the points above.

Software and code

Policy information about availability of computer code

Data collection	The data consist of 11 antibody titer measurements on 24,402 individuals. These were organized and assembled into a database using Matlab scripts (all methods described in Nhat et al, Nat Sci Rep, 7:6060, 2017). Data are available at https://github.com/bonilab/seroepi-02FL-influenza-vietnam-PCA
Data analysis	All analysis was done in Matlab version 2019b. Analyses performed were principal components analysis, and likelihood optimization using a Nelder-Mead routine in order to infer attack rate. Likelihood profiling (used to obtain confidence intervals for annual attack rate) was also done using Matlab's Nelder-Mead routine.

For manuscripts utilizing custom algorithms or software that are central to the research but not yet described in published literature, software must be made available to editors and reviewers. We strongly encourage code deposition in a community repository (e.g. GitHub). See the Nature Research guidelines for submitting code & software for further information.

Data

Policy information about availability of data

All manuscripts must include a data availability statement. This statement should provide the following information, where applicable:

- Accession codes, unique identifiers, or web links for publicly available datasets
- A list of figures that have associated raw data
- A description of any restrictions on data availability

Data and code are posted publicly at https://github.com/bonilab/seroepi-02FL-influenza-vietnam-PCA

Field-specific reporting

Please select the one below that is the best fit for your research. If you are not sure, read the appropriate sections before making your selection.

[x] Life sciences [ ] Behavioural & social sciences [ ] Ecological, evolutionary & environmental sciences

For a reference copy of the document with all sections, see nature.com/documents/nr-reporting-summary-flat.pdf

Life sciences study design

All studies must disclose on these points even when the disclosure is negative.

Sample size	The primary purpose of designing our serum bank as a serological time series was to infer epidemic patterns in the population using serological data only. 200 samples collected every two months was deemed the most statistically appropriate collection time and frequency (Vinh and Boni, Epidemics, 12:30-39, 2015) in order to infer once-yearly epidemic patterns. For the present analysis, all samples from these collections were used since all were available. No sample size calculation was done to determine what size sub-sample would be sufficient for the principal components analysis, as all samples were already processed and all data were available.
Data exclusions	Individuals missing one of the 11 titer measurements were excluded. This reduced the sample collection from ~35K individuals to ~24K individuals.
Replication	Replicability of titer measurements was assessed by assaying 32 samples in 11-tuplicate (i.e. 11 x 32 assays were done on 32 samples). Standard deviation on log-titer scale was around 0.6, which is an improvement in replicability over HAI and MN assays. This was published in 2017 (Nhat et al, 7:6060, Nat Sci Rep, section 3 in supplement).
Randomization	There was no intervention group in this study, and no randomization or blinding was required.
Blinding	There was no intervention group in this study, and no randomization or blinding was required.

Reporting for specific materials, systems and methods

We require information from authors about some types of materials, experimental systems and methods used in many studies. Here, indicate whether each material, system or method listed is relevant to your study. If you are not sure if a list item applies to your research, read the appropriate section before selecting a response.

Materials & experimental systems

n/a	Involved in the study	
[x]	[ ]	Antibodies
[x]	[ ]	Eukaryotic cell lines
[x]	[ ]	Palaeontology and archaeology
[x]	[ ]	Animals and other organisms
[ ]	[x]	Human research participants
[x]	[ ]	Clinical data
[x]	[ ]	Dual use research of concern

Methods

n/a	Involved in the study	
[x]	[ ]	ChIP-seq
[x]	[ ]	Flow cytometry
[x]	[ ]	MRI-based neuroimaging

Human research participants

Policy information about studies involving human research participants

Population characteristics	Hospital-going population in 10 cities in Vietnam. Samples were collected from biochemistry and haematology labs of 10 hospitals, for inpatient and outpatient visits, where the hospital visit could be of any indication sufficient to warrant a blood panel or blood draw for other reasons. HIV+ patients were excluded. The patient covariates included in the data are (1) age, (2) gender, (3) outpatient status, and (4) admitting ward. No covariates on current diagnosis or treatment are available.
Recruitment	No recruitment of patients. Only collection of residual serum samples from biochemistry/haematology labs. Study samples are completely de-linked from patient records.
Ethics oversight	The study was approved by the Scientifc and Ethical Committee of the Hospital for Tropical Diseases in Ho Chi Minh City and the Oxford Tropical Research Ethics Committee at the University of Oxford.

Note that full information on the approval of the study protocol must also be provided in the manuscript.
